# GENCODE: reference annotation for the human and mouse genomes in 2023

**DOI:** 10.1093/nar/gkac1071

**Published:** 2022-11-24

**Authors:** Adam Frankish, Sílvia Carbonell-Sala, Mark Diekhans, Irwin Jungreis, Jane E Loveland, Jonathan M Mudge, Cristina Sisu, James C Wright, Carme Arnan, If Barnes, Abhimanyu Banerjee, Ruth Bennett, Andrew Berry, Alexandra Bignell, Carles Boix, Ferriol Calvet, Daniel Cerdán-Vélez, Fiona Cunningham, Claire Davidson, Sarah Donaldson, Cagatay Dursun, Reham Fatima, Stefano Giorgetti, Carlos Garcıa Giron, Jose Manuel Gonzalez, Matthew Hardy, Peter W Harrison, Thibaut Hourlier, Zoe Hollis, Toby Hunt, Benjamin James, Yunzhe Jiang, Rory Johnson, Mike Kay, Julien Lagarde, Fergal J Martin, Laura Martínez Gómez, Surag Nair, Pengyu Ni, Fernando Pozo, Vivek Ramalingam, Magali Ruffier, Bianca M Schmitt, Jacob M Schreiber, Emily Steed, Marie-Marthe Suner, Dulika Sumathipala, Irina Sycheva, Barbara Uszczynska-Ratajczak, Elizabeth Wass, Yucheng T Yang, Andrew Yates, Zahoor Zafrulla, Jyoti S Choudhary, Mark Gerstein, Roderic Guigo, Tim J P Hubbard, Manolis Kellis, Anshul Kundaje, Benedict Paten, Michael L Tress, Paul Flicek

**Affiliations:** European Molecular Biology Laboratory, European Bioinformatics Institute, Wellcome Genome Campus, Hinxton, Cambridge CB10 1SD, UK; Department of Bioinformatics and Genomics, Centre for Genomic Regulation (CRG), The Barcelona Institute of Science andTechnology, Dr. Aiguader 88, Barcelona 08003, Catalonia, Spain; UC Santa Cruz Genomics Institute, University of California, Santa Cruz, CA 95064, USA; MIT Computer Science and Artificial Intelligence Laboratory, 32 Vassar St, Cambridge, MA 02139,USA; Broad Institute of MIT and Harvard, 415 Main Street, Cambridge, MA 02142, USA; European Molecular Biology Laboratory, European Bioinformatics Institute, Wellcome Genome Campus, Hinxton, Cambridge CB10 1SD, UK; European Molecular Biology Laboratory, European Bioinformatics Institute, Wellcome Genome Campus, Hinxton, Cambridge CB10 1SD, UK; Department of Molecular Biophysics and Biochemistry, Yale University, New Haven, CT 06520, USA; Department of Life Sciences, Brunel University London, Uxbridge UB8 3PH, UK; Functional Proteomics, Division of Cancer Biology, Institute of Cancer Research, 237 Fulham Road, London SW3 6JB, UK; Department of Bioinformatics and Genomics, Centre for Genomic Regulation (CRG), The Barcelona Institute of Science andTechnology, Dr. Aiguader 88, Barcelona 08003, Catalonia, Spain; European Molecular Biology Laboratory, European Bioinformatics Institute, Wellcome Genome Campus, Hinxton, Cambridge CB10 1SD, UK; Department of Genetics, Stanford University, Palo Alto, CA, USA; Department of Computer Science, Stanford University, Palo Alto, CA, USA; European Molecular Biology Laboratory, European Bioinformatics Institute, Wellcome Genome Campus, Hinxton, Cambridge CB10 1SD, UK; European Molecular Biology Laboratory, European Bioinformatics Institute, Wellcome Genome Campus, Hinxton, Cambridge CB10 1SD, UK; European Molecular Biology Laboratory, European Bioinformatics Institute, Wellcome Genome Campus, Hinxton, Cambridge CB10 1SD, UK; MIT Computer Science and Artificial Intelligence Laboratory, 32 Vassar St, Cambridge, MA 02139,USA; Broad Institute of MIT and Harvard, 415 Main Street, Cambridge, MA 02142, USA; Department of Bioinformatics and Genomics, Centre for Genomic Regulation (CRG), The Barcelona Institute of Science andTechnology, Dr. Aiguader 88, Barcelona 08003, Catalonia, Spain; Bioinformatics Unit, Spanish National Cancer Research Centre (CNIO), Calle Melchor Fernandez Almagro, 3, 28029 Madrid, Spain; European Molecular Biology Laboratory, European Bioinformatics Institute, Wellcome Genome Campus, Hinxton, Cambridge CB10 1SD, UK; European Molecular Biology Laboratory, European Bioinformatics Institute, Wellcome Genome Campus, Hinxton, Cambridge CB10 1SD, UK; European Molecular Biology Laboratory, European Bioinformatics Institute, Wellcome Genome Campus, Hinxton, Cambridge CB10 1SD, UK; Department of Molecular Biophysics and Biochemistry, Yale University, New Haven, CT 06520, USA; Program in Computational Biology and Bioinformatics, Yale University, New Haven, CT 06520, USA; European Molecular Biology Laboratory, European Bioinformatics Institute, Wellcome Genome Campus, Hinxton, Cambridge CB10 1SD, UK; European Molecular Biology Laboratory, European Bioinformatics Institute, Wellcome Genome Campus, Hinxton, Cambridge CB10 1SD, UK; European Molecular Biology Laboratory, European Bioinformatics Institute, Wellcome Genome Campus, Hinxton, Cambridge CB10 1SD, UK; European Molecular Biology Laboratory, European Bioinformatics Institute, Wellcome Genome Campus, Hinxton, Cambridge CB10 1SD, UK; European Molecular Biology Laboratory, European Bioinformatics Institute, Wellcome Genome Campus, Hinxton, Cambridge CB10 1SD, UK; European Molecular Biology Laboratory, European Bioinformatics Institute, Wellcome Genome Campus, Hinxton, Cambridge CB10 1SD, UK; European Molecular Biology Laboratory, European Bioinformatics Institute, Wellcome Genome Campus, Hinxton, Cambridge CB10 1SD, UK; European Molecular Biology Laboratory, European Bioinformatics Institute, Wellcome Genome Campus, Hinxton, Cambridge CB10 1SD, UK; European Molecular Biology Laboratory, European Bioinformatics Institute, Wellcome Genome Campus, Hinxton, Cambridge CB10 1SD, UK; MIT Computer Science and Artificial Intelligence Laboratory, 32 Vassar St, Cambridge, MA 02139,USA; Broad Institute of MIT and Harvard, 415 Main Street, Cambridge, MA 02142, USA; Program in Computational Biology and Bioinformatics, Yale University, New Haven, CT 06520, USA; Department of Medical Oncology, Bern University Hospital, Murtenstrasse 35, 3008 Bern, Switzerland; School of Biology and Environmental Science, University College Dublin, Belfield, Dublin 4, D04 V1W8, Ireland; European Molecular Biology Laboratory, European Bioinformatics Institute, Wellcome Genome Campus, Hinxton, Cambridge CB10 1SD, UK; Department of Bioinformatics and Genomics, Centre for Genomic Regulation (CRG), The Barcelona Institute of Science andTechnology, Dr. Aiguader 88, Barcelona 08003, Catalonia, Spain; European Molecular Biology Laboratory, European Bioinformatics Institute, Wellcome Genome Campus, Hinxton, Cambridge CB10 1SD, UK; Bioinformatics Unit, Spanish National Cancer Research Centre (CNIO), Calle Melchor Fernandez Almagro, 3, 28029 Madrid, Spain; Department of Genetics, Stanford University, Palo Alto, CA, USA; Department of Computer Science, Stanford University, Palo Alto, CA, USA; Department of Molecular Biophysics and Biochemistry, Yale University, New Haven, CT 06520, USA; Program in Computational Biology and Bioinformatics, Yale University, New Haven, CT 06520, USA; Bioinformatics Unit, Spanish National Cancer Research Centre (CNIO), Calle Melchor Fernandez Almagro, 3, 28029 Madrid, Spain; Department of Genetics, Stanford University, Palo Alto, CA, USA; Department of Computer Science, Stanford University, Palo Alto, CA, USA; European Molecular Biology Laboratory, European Bioinformatics Institute, Wellcome Genome Campus, Hinxton, Cambridge CB10 1SD, UK; European Molecular Biology Laboratory, European Bioinformatics Institute, Wellcome Genome Campus, Hinxton, Cambridge CB10 1SD, UK; Department of Genetics, Stanford University, Palo Alto, CA, USA; Department of Computer Science, Stanford University, Palo Alto, CA, USA; European Molecular Biology Laboratory, European Bioinformatics Institute, Wellcome Genome Campus, Hinxton, Cambridge CB10 1SD, UK; European Molecular Biology Laboratory, European Bioinformatics Institute, Wellcome Genome Campus, Hinxton, Cambridge CB10 1SD, UK; European Molecular Biology Laboratory, European Bioinformatics Institute, Wellcome Genome Campus, Hinxton, Cambridge CB10 1SD, UK; European Molecular Biology Laboratory, European Bioinformatics Institute, Wellcome Genome Campus, Hinxton, Cambridge CB10 1SD, UK; Computational Biology of Noncoding RNA, Institute of Bioorganic Chemistry, Polish Academy of Sciences, Noskowskiego 12/14, 61-704 Poznan, Poland; European Molecular Biology Laboratory, European Bioinformatics Institute, Wellcome Genome Campus, Hinxton, Cambridge CB10 1SD, UK; Department of Molecular Biophysics and Biochemistry, Yale University, New Haven, CT 06520, USA; Institute of Science and Technology for Brain-Inspired Intelligence, Fudan University, Shanghai 200433, China; European Molecular Biology Laboratory, European Bioinformatics Institute, Wellcome Genome Campus, Hinxton, Cambridge CB10 1SD, UK; Department of Genetics, Stanford University, Palo Alto, CA, USA; Department of Computer Science, Stanford University, Palo Alto, CA, USA; Functional Proteomics, Division of Cancer Biology, Institute of Cancer Research, 237 Fulham Road, London SW3 6JB, UK; Department of Molecular Biophysics and Biochemistry, Yale University, New Haven, CT 06520, USA; Program in Computational Biology and Bioinformatics, Yale University, New Haven, CT 06520, USA; Department of Bioinformatics and Genomics, Centre for Genomic Regulation (CRG), The Barcelona Institute of Science andTechnology, Dr. Aiguader 88, Barcelona 08003, Catalonia, Spain; Departament de Ciències Experimentals i de la Salut, Universitat Pompeu Fabra (UPF), Barcelona, E-08003 Catalonia, Spain; Department of Medical and Molecular Genetics, King's College London, Guys Hospital, Great Maze Pond, London SE1 9RT, UK; MIT Computer Science and Artificial Intelligence Laboratory, 32 Vassar St, Cambridge, MA 02139,USA; Broad Institute of MIT and Harvard, 415 Main Street, Cambridge, MA 02142, USA; Department of Genetics, Stanford University, Palo Alto, CA, USA; Department of Computer Science, Stanford University, Palo Alto, CA, USA; UC Santa Cruz Genomics Institute, University of California, Santa Cruz, CA 95064, USA; Bioinformatics Unit, Spanish National Cancer Research Centre (CNIO), Calle Melchor Fernandez Almagro, 3, 28029 Madrid, Spain; European Molecular Biology Laboratory, European Bioinformatics Institute, Wellcome Genome Campus, Hinxton, Cambridge CB10 1SD, UK

## Abstract

GENCODE produces high quality gene and transcript annotation for the human and mouse genomes. All GENCODE annotation is supported by experimental data and serves as a reference for genome biology and clinical genomics. The GENCODE consortium generates targeted experimental data, develops bioinformatic tools and carries out analyses that, along with externally produced data and methods, support the identification and annotation of transcript structures and the determination of their function. Here, we present an update on the annotation of human and mouse genes, including developments in the tools, data, analyses and major collaborations which underpin this progress. For example, we report the creation of a set of non-canonical ORFs identified in GENCODE transcripts, the LRGASP collaboration to assess the use of long transcriptomic data to build transcript models, the progress in collaborations with RefSeq and UniProt to increase convergence in the annotation of human and mouse protein-coding genes, the propagation of GENCODE across the human pan-genome and the development of new tools to support annotation of regulatory features by GENCODE. Our annotation is accessible via Ensembl, the UCSC Genome Browser and https://www.gencodegenes.org.

## INTRODUCTION

GENCODE builds reference quality human and mouse annotation of protein-coding genes, long non-coding RNAs (lncRNAs) and small non-coding RNAs (sRNAs), as well as pseudogenes of protein-coding genes and immunoglobulin and T-cell receptor segments (1,2). GENCODE annotation is rich in its representation of alternative splicing within protein-coding and lncRNA loci, its assignment of ‘biotypes’ describing biological function at the transcript level, and the usage of ‘attributes’ to add further information (3,4). We make all our annotation freely available to support genome interpretation and biomedical research. The GENCODE consortium also produces targeted transcriptomic data, and develops and refines bioinformatic tools and analysis for the annotation of genes and transcripts. The work of the consortium drives updates to genes of every biotype, including (i) the identification of novel protein-coding genes, lncRNAs and pseudogenes; (ii) capturing novel alternatively spliced transcripts at protein-coding and lncRNA loci; (iii) the iterative reassessment of existing genes and transcripts that can lead to their update or removal; (iv) the identification and integration of novel biological features into the annotation. In addition, GENCODE is developing methods and workflows for the annotation of regulatory features and the physical connections between them.

The progress made by GENCODE over the last two years includes the migration of mouse gene annotation from GRCm38 to the new mouse reference assembly GRCm39 and the update of 44 protein-coding genes that were only present or intact on the new assembly. Mouse also benefited from the first application of manually supervised automated annotation of lncRNAs using data generated by the Capture long-seq (CLS) method. We have continued joint efforts with RefSeq (5) and UniProt (6) to drive towards convergence on the annotation of protein-coding genes. We have essentially completed the project to annotate a single representative ‘MANE Select’ transcript for every human protein-coding gene as part of the Matched Annotation from NCBI and EMBL-EBI (MANE) collaboration (7), thus ensuring that its structure and sequence is identical in the GENCODE and RefSeq genesets. As part of a community driven effort to support the annotation of ORFs whose translation has been validated by experimental data but whose protein-coding potential is uncertain (8) we have created the first set of Ribo-seq ORFs linked to GENCODE genes and transcripts and distributed it via gencodegenes.org. Similarly, we have played a leading role in the Long-read RNA-seq Genome Annotation Assessment Project (LRGASP) (9) to identify the state of the art in the computational creation of transcript models from long transcriptomic data and their quantification. We have also worked to map GENCODE genes to newly sequenced, high quality human genome assemblies produced by the Telomere-to-Telomere (T2T) (10) and Human Pangenome Reference Consortium (HPRC) (11) consortia to provide high quality reference annotation. We have also started to develop computational tools to support the annotation of regulatory features within GENCODE, allowing us to move towards the annotation of an extended model of the gene, including its associated regulatory features.

## GENE ANNOTATION

The GENCODE consortium has improved and extended the annotation of the human and mouse reference genomes, producing seven human (GENCODE 35–41) and seven mouse (M24–M30) GENCODE releases since June 2020, with M26 being the first release on the GRCm39 mouse assembly. Table 1 contains annotation statistics from the most recent GENCODE releases. For both species, the addition of new transcript models remains the dominant source of change between releases. To a large extent, the increases in transcript counts reflect our continued development of the TAGENE pipeline, which we devised to aid the incorporation of long-read datasets—including our in-house CLS data—into transcript models. Following iterative testing led by manual annotators, we have implemented the use of the tmerge pipeline (https://github.com/julienlag/tmerge) within TAGENE as a replacement for PASA (12) for the clustering of reads into prospective transcript models; having tested over 1000 TAGENE models, we found tmerge to produce notably fewer clustering errors.

**Table 1.** Total numbers of genes and transcripts in the GENCODE 41 (human) and GENCODE M30 (mouse) releases by gene functional biotype. Readthrough loci that span multiple individual protein-coding genes are excluded from this count

			Protein-coding	lncRNA	Pseudogene	sRNA	IG/TR
Human	GENCODE 41	Genes	19370	19095	14737	7566	410
		Transcripts	167599	54291	19737	7566	420
Mouse	GENCODE M30	Genes	21668	14525	13468	6105	494
		Transcripts	101716	25419	15026	6105	656

Since June 2020, ∼37 000 genes (∼27 000 human and ∼10 000 mouse) and ∼61 000 transcripts (∼49 000 human and ∼11 000 mouse) have either been created or updated in the GENCODE geneset. Table 2 contains a breakdown of new and updated genes and transcripts by their functional biotype.

**Table 2.** Numbers of genes and transcripts that have been added to or updated in GENCODE human and mouse annotation since June 2020

		Human New	Updated	New and updated	Mouse New	Updated	New and updated
	Protein-coding	179	10539	10718	133	3960	4093
Genes	lncRNA	2448	7344	9792	1580	2711	4291
	Pseudogene	23	6065	6088	40	2101	2141
	**Total**	**2650**	**23948**	**26598**	**1753**	**8772**	**10428**
	Protein-coding	20296	8972	29268	1603	553	2156
Transcripts	lncRNA	6233	3997	10230	6603	1953	8556
	Pseudogene	1553	1338	2891	542	200	742
	**Total**	**28082**	**20978**	**49060**	**8748**	**2706**	**11454**

Although the primary annotation effort has been concentrated on the human genome, we have lifted-over mouse gene annotation from GRCm38 to the new mouse reference genome assembly GRCm39, checking and updating genes that were not mapped correctly and annotating 44 new genes. In addition, this period has seen the first manually supervised automated annotation of lncRNA genes and transcripts in mouse using the TAGENE pipeline. TAGENE updates alone added 1573 novel genes and updated a further 1479. In total, 6591 novel transcripts were added and 1824 updated (almost all of which were extensions to former partial transcripts).

While the headline counts of protein-coding genes remain relatively stable for both human and mouse, the actual protein-coding gene set has been subject to continual review and update over this period. In human, 90 completely new protein-coding genes have been identified and annotated, with 30 pre-existing protein-coding genes deleted; in mouse 36 were added and 49 removed. In addition, another 89 human genes have been made protein-coding that were previously annotated as other functional biotypes (97 in mouse), while 63 human protein-coding genes have been reannotated as other functional biotypes (135 in mouse). LncRNAs continue to show the largest increase in number at the gene level in both human and mouse, and at the transcript level in mouse. This reflects our ability to use TAGENE to add high confidence transcript models based on targeted sequencing experiments, especially CLS (13).

GENCODE continues active collaborations with other reference annotation databases, especially in order to drive convergence in the annotation of protein-coding genes in human and mouse. Such work also identifies loci for which convergence is not yet possible. The MANE (7) collaboration was initiated to annotate an agreed transcript (labelled as MANE_Select) for every human protein-coding gene. All MANE transcripts are a 100% match for sequence and structure (splicing, UTR and CDS) in both the Ensembl/GENCODE and RefSeq annotation sets. We have achieved the essential completion of the first phase of the project: to annotate a MANE Select transcript at every protein-coding gene on the human reference genome. This has been released as MANE v1.0. Over the last 2 years ∼3000 MANE Select transcripts have been annotated, increasing coverage to 99.1% of human protein coding genes and 99.8% of clinical genes. In addition, we have annotated 58 MANE Plus Clinical transcript models, which are alternatively spliced transcripts within genes where a single transcript does not support the description of all known and likely pathogenic variants in ClinVar(14).

We work with the UniProt project to improve the compatibility of our annotations of human and mouse protein-coding genes and proteins via the Genome Integration with FuncTion and Sequence (GIFTS) project (https://www.ebi.ac.uk/gifts). UniProt proteins that do not map to Ensembl genes are flagged for investigation, with an additional ∼500 human and ∼400 mouse UniProt accessions undergoing manual review. In addition, the protein translations of MANE Select transcripts have been compared with UniProt canonical isoforms, with 267 MANE Select mismatches identified, most which have now been updated by UniProt to match GENCODE annotation.

In collaboration with external partners, GENCODE launched LRGASP (Long-Read Genome Annotation Assessment Project) (9), a challenge-style experiment to examine the utility of–and to identify current best practice for–long-read transcriptomic methodologies for transcript annotation and quantification. GENCODE has led or contributed to project planning, the management of data submission, data visualisation and analysis, the generation of novel CapTrap_PacBio and CapTrap_ONT datasets, and the experimental evaluation of submitted transcripts. In particular, we manually created reference annotation of ∼1300 transcripts supported by reads generated by the LRGASP consortium; these are being used as ‘ground truth’ in the assessment of transcript models submitted by participants.

## PROTEIN-CODING GENES

Ribosome profiling (Ribo-seq) experiments identify many thousands of translated open reading frames (ORFs) within what were previously believed to be untranslated regions of protein-coding genes and lncRNAs. These ORFs are of increasing interest due to their potential to encode unannotated functional proteins, and they can also represent aberrant translations limited to disease states. Furthermore, translation can also impart function at the regulatory level. Upstream ORFs (uORFs) are of particular interest due to their suspected widespread occurrence in 5′ UTRs, as well as their implication in disease whereby disrupting variants can affect the translation of the downstream canonical CDS. The creation of new uORFs by gain of function variants may also affect downstream translation. Ribo-seq ORFs have previously not been included in reference gene catalogues, which hinders their investigation and characterisation. To address this, a community-led effort organised by GENCODE and supported by HGNC (15), UniProt and HUPO has produced an initial consolidated catalogue of 7264 annotated human Ribo-seq ORFs without functional characterisation (8). GENCODE released this catalogue mapped to GENCODE 35. The GENCODE Ribo-seq ORF set has now been taken forward by PeptideAtlas (16) on behalf of HUPO (https://hupo.org/) in order to examine their protein-coding potential with proteomics data.

**Figure 1.**
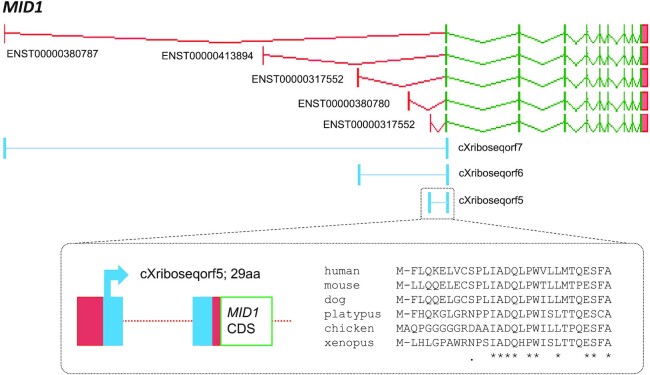
Upstream open reading frames in *MID1*. GENCODE 41 annotation includes five distinct transcript start site regions within midline 1 (*MID1*), a TRIM-family protein-coding gene. Five representative transcripts are shown here; additional transcriptional complexity has been omitted for clarity. Three replicated Ribo-seq ORFs are located on three transcripts: cXriboseqorf7, cXriboseqorf6 and cXriboseqorf5 found respectively on ENST00000380787, ENST00000317552 (the MANE Select transcript) and ENST00000317552. Each is a translated uORF, with three distinct first exon ORF portions being linked to a shared second exon ORF portion. The shared ORF portion has a positive PhyloCSF score, indicating that it has evolved as a protein-coding sequence. However, PhyloCSF only supports the protein-coding potential of one of the three alternative first exons, cXriboseqorf5 on ENST00000317552. A multispecies protein alignment (inset) finds that cXriboseqorf5 has intact orthologs across tetrapods with accompanying transcript support; beyond the five representative species shown, the ORF appears potentially conserved across vertebrates. This ORF has thus been annotated as the new protein-coding gene ENSG00000291314. In contrast, the first exon ORF portions of cXriboseqorf7 and cXriboseqorf6 present equivocal evolutionary signatures, lacking PhyloCSF support to indicate protein-level function. Nonetheless, cXriboseqorf7 at least is conserved as an ORF in mammals as well as reptiles and avians, and if this ORF is not protein-coding it may turn out to have a regulatory function that is evolving under a different mode of selection. This may also be true of cXriboseqorf6, and in fact, we do not rule out the possibility that both cXriboseqorf7 and cXriboseqorf6 encode functional proteins in spite of the lack of PhyloCSF support.

We have used PhyloCSF (17) to assess the protein-coding potential of over 230 000 novel unannotated ORFs found by analysis of Ribo-seq data alongside another analysis of mass spectrometry of MHC Class I-bound peptides (18). While the vast majority of these ORFs were found not to be under detectable constraint at the protein level, ∼70 were shown to include regions possessing strong signals of evolution as all or part of protein-coding genes, including a translated uORF within *MID1* (Figure 1). This work has led to the annotation of novel protein-coding regions, including complete ORFs as CDS, as well the annotation of N-terminus CDS extensions, alternative 3' ends, and exon extensions in the GENCODE annotation.

We have developed a process to identify potentially important missing transcript models within protein coding genes. Based on an integrated analysis of RefSeq and UniProtKB annotations, predictions from publications, peptides from large-scale proteomics experiments, human variation data (19), conflicting structural and functional data in the APPRIS database (20), and evolutionary history of coding exons (21,22), we have identified 895 putative missing human transcripts with 441 added during ongoing manual review. We have developed TRIFID (https://github.com/fpozoc/trifid), a machine learning model based on cross-species conservation, GENCODE annotations, protein structural and functional features and experimental evidence that predicts the functional importance of the protein isoforms encoded by GENCODE transcripts (23). TRIFID v1.0.0 has been incorporated into the updated APPRIS database of principal splice isoforms (24) from 2020_06.v31.

GENCODE annotation continues to use proteomics data to validate predictions of protein-coding potential and identify unannotated protein-coding genes. We have generated synthetic peptides for targeted validation and quantification of proteins, developing a pipeline to select peptides with the highest specificity and mass spectrometry viability for synthesis. Experimental data has been used to build spectral libraries to identify peptides in large sets of public spectra in the PRIDE (25) repository, finding 88 peptides with a significant identification.

We have continued to upgrade infrastructure supporting protein-coding annotation, updating tools to work with new alignments and assemblies. For human we have integrated the 241-mammal zoonomia alignment (26) into the CodAlignView tool (https://data.broadinstitute.org/compbio1/CodAlignViewUsersGuide.html), leveraging its more comprehensive genome alignment for assessing protein-coding potential of ambiguous human exons and short ORFs. For the mouse, we have incorporated the GRCm39 assembly using the 35 vertebrate alignment into CodAlignView and created PhyloCSF browser tracks for the UCSC(27) and Ensembl (28) Genome Browsers. We have further developed PhyloCSF-Psi (https://github.com/mlin/PhyloCSF/wiki), a version of PhyloCSF that adjusts for region length, and enhanced CodAlignView to better handle alignment gaps, to display phylogenetic trees, and to provide better feedback about splice site predictions. We have updated the PhyloCSF Candidate Coding Region list for GENCODE versions 35–41 and M24–30, to help guide the HAVANA expert-annotation team to the most promising genomic regions for new protein-coding and pseudogene annotations. We also applied our comparative tools to improve annotation of genes in the SARS-CoV-2 virus (29,30), in the process improving methods for distinguishing overlapping protein-coding ORFs, which are common in viruses and also present in some human and mouse genes.

### lncRNAS

In addition to generating CapTrap ONT and PacBio Sequel II datasets for LRGASP, we have developed and tested two broad custom RNA capture designs with the goal of expanding human and mouse lncRNA gene and transcript annotations. These designs, designated Hv3 and Mv2, target 80 Mb and 64 Mb, respectively in human and mouse. These panels target a large number of GENCODE-unannotated elements in external ncRNA catalogues such as Bigtranscriptome (31), FANTOMCat (32) and miTranscriptome (33) and computational predictions. We prepared pre- and post-CLS capture libraries, generating Illumina Hi-Seq, PacBio Sequel II and ONT sequence from 22 human tissues, 6 human cell lines, 20 mouse tissues and 1 mouse cell line, which is analysed using an updated LyRic workflow (https://github.com/julienlag/LyRic) and fed into downstream annotation processes.

## PSEUDOGENES

We have continued to evaluate the coding potential and conservation of putative unitary pseudogenes to generate candidate lists for manual review. We have investigated both the original loss of function event and the most recent speciation event preserving the functional homolog, along with the transcriptional activity and selection profile of putative unitary pseudogenes. For mouse we have used the 241-mammal Zoonomia alignment to compile a list of candidate unannotated mouse unitary pseudogenes with functional orthologs in human(26). We have extended our pseudogene annotation in non-human primates (NHPs), systematically annotating the processed and duplicated pseudogenes for 15 species using their latest high-quality assemblies. We then identified the human-specific pseudogenes via a comparative analysis and applied a computational framework to accurately estimate the expression of the pseudogenes in human and macaque brain. We identified a set of tissue-specific and region-specific pseudogenes and showed that they exhibit higher tissue and regional specificity than protein-coding genes. Finally, we observed different patterns of expression changes in psychiatric disorders between pseudogenes and protein-coding genes.

## THE HUMAN PANGENOME

**Figure 2.**
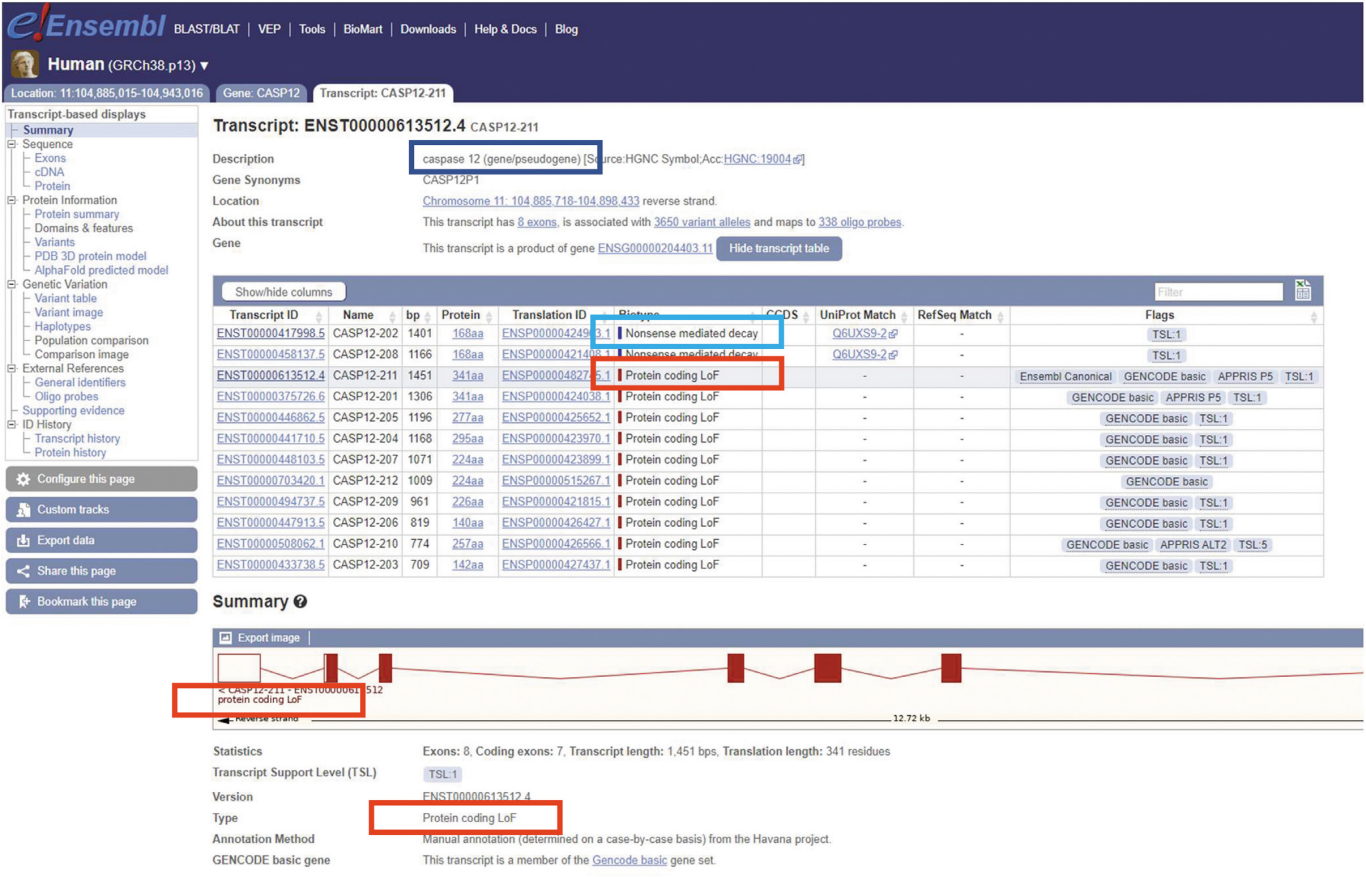
Screenshot from the Ensembl genome browser of the transcript view page for the gene *CASP12* which contains transcripts annotated as Protein coding LoF. The status of the gene as having both functional and non-functional alleles is indicated by the dark blue box. The annotation of nonsense-mediated decay transcripts with fixed premature stop codons is indicated by the light blue box and the locations of the Protein coding LoF biotype flag are highlighted by the red box.

We have participated in the annotation of the developing human pangenome (e.g. 34), supporting the annotation of the T2T consortium's CHM13 human cell line assembly. Using human GENCODE transcripts as a reference, CHM13 was annotated with the Comparative Annotation Toolkit (CAT) (35) combined with LiftOff (36). GENCODE is also actively participating in the HPRC, providing an initial set of population gene annotations on 47 high-quality diploid human genome assemblies. We have provided one gene annotation set using CAT with GENCODE as the reference gene set and assisted in the creation of a second GENCODE-derived annotation set by Ensembl (11). Annotation of the pangenome creates different alleles of the same gene on different assemblies and it is essential that these different alleles can be easily identified, whether they represent functional or non-functional copies. Historically GENCODE has annotated protein-coding genes with loss-of-function (LoF) mutations on the reference genome as polymorphic pseudogenes (3). From GENCODE 40 onwards we have updated their annotation to better reflect their status in the pangenome; these loci are now annotated as protein-coding genes (rather than pseudogenes) with their transcripts flagged as ‘protein-coding LoF’. The identification of these transcripts in the Ensembl browser is shown in Figure 2.

## REGULATORY FEATURE ANNOTATION

GENCODE aims to broaden the annotation of genes to include the proximal and distal regulatory elements that control their expression. We will annotate the ‘extended gene’ where transcript models are connected to annotated promoter features and promoters connected to enhancer annotation. To this end, we have developed computational methods that utilise nascent transcriptomic data to identify and define regulatory regions and their connections to genes. We have been developing deep learning models of nascent transcription assays such as PRO-seq, GRO-seq and PRO-cap in order to decipher the cis-regulatory syntax of transcription initiation at single base resolution. We extended BPNet (37) neural networks to accurately map DNA sequence to base-resolution profiles from ENCODE PRO-cap experiments and applied the DeepLIFT (38) feature attribution method to these trained models to infer the importance/contribution scores of individual bases to model's predictions of profiles in each peak sequence across the genome. We then summarised predictive motif patterns across all peaks using TF-Modisco (https://github.com/kundajelab/tfmodisco), which learns a non-redundant set of motifs from predictive subsequences across all PRO-cap peak regions. We have also used EpiMap (39), an integrative epigenomic reference map of human cells and tissues to annotate high-resolution gene-regulatory regions and their tissue-specific target genes, enhancing each gene annotation with its regulatory elements. These links were further extended using single-cell accessibility information (scATAC-seq) and the integration of coding and non-coding annotations with disease associated variation. Candidate regulatory regions and connections defined by this approach will be manually reviewed and the results fed back into the automatic annotation process.

## DATA ACCESS

GENCODE gene sets are currently updated up to four times each year for both human and mouse. Each release is versioned and made available immediately upon release from Ensembl and https//www.gencodegenes.org with release on the UCSC Genome Browser normally following shortly thereafter. The current human release is GENCODE 41 (July 2022) and the current mouse release is GENCODE M30 (July 2022). Additional information and previous releases can be found at https//www.gencodegenes.org.

GENCODE is the now the standardised default human and mouse annotation for both the Ensembl and UCSC genome browsers. Data is presented through all of the standard interfaces from both resources.

To expedite public access to updated annotation between releases, all annotation changes are made freely available within 24 h via the ‘GENCODE update’ Track Hub, which can be accessed at both the Ensembl and UCSC genome browsers. In the Ensembl browser, the hub has been added to the Track Hub Registry (accessed via the ‘Custom tracks’ section), and can be connected to by searching for ‘GENCODE update’. Alternatively, the data can be added as a custom track in both Ensembl and UCSC browsers (http://ftp.ebi.ac.uk/pub/databases/gencode/update_trackhub/hub.txt).

We have made available the public ‘Synonymous Constraint’ track hub in the UCSC Genome Browser that shows protein-coding regions under synonymous constraint, indicating an overlapping function, and synonymous accelerated regions, indicating a high mutation rate (https://data.broadinstitute.org/compbio1/SynonymousConstraintTracks/trackHub/). PhyloCSF and APPRIS Principal Splice Isoforms are also available via UCSC’s Public Hubs.

Other interfaces include the Ensembl FTP site (ftp://ftp.ensembl.org/pub/), which includes gene sets in GFF3, Genbank and GTF formats and full download of the complete Ensembl databases. More complex and customizable gene set queries can be created via the Ensembl Biomart (https://www.ensembl.org/biomart/). Biomart also allows queries based on MANE annotation.

Programmatic access to the GENCODE gene sets is possible via the extensive Ensembl Perl API and the language-agnostic Ensembl REST API (40). Programmatic access facilitates advanced genome-wide analysis such as retrieval of supporting features and associated gene trees. Examples of REST endpoint usage and starter scripts in different languages are at https://rest.ensembl.org.

GENCODE-specific training materials and GENCODE focused workshops from the Ensembl Outreach team are available via the Ensembl Training portal (http://training.ensembl.org).

## CONCLUSION

The GENCODE consortium aspires to drive improvement in the gene annotation of the human and mouse reference and pan-genomes. We continue to develop new computational and manual approaches to leverage increasing quality and volume of transcriptomic data and integrate it with orthogonal data and analysis to increase the breadth and depth of our annotation. Our targeted generation of long transcriptomic data—for example at lncRNA genes - combined with use of public data to the same end for alternatively spliced transcripts of protein-coding genes supports our progress towards a more complete catalogue of genes and transcripts. This is assisted by our participation in community projects, like LRGASP, that investigate the best ways to utilise transcriptomic data and, like the Ribo-seq consortium, work to identify and annotate the novel functional features they encode. Ultimately, the extension of the gene model to include proximal and distal regulatory features will provide the gene and its transcripts with more complete contextual information that will support detailed downstream analysis.

## DATA AVAILABILITY

No new data were generated or analysed in support of this research.
